# Endocrine and metabolic interactions in healthy pregnancies and hyperinsulinemic pregnancies affected by polycystic ovary syndrome, diabetes and obesity

**DOI:** 10.3389/fendo.2022.993619

**Published:** 2023-01-17

**Authors:** Adriana C. H. Neven, Aya Mousa, Jacqueline A. Boyle, Helena J. Teede

**Affiliations:** ^1^ Monash Centre for Health Research and Implementation (MCHRI), School of Public Health and Preventive Medicine, Monash University, Clayton, VIC, Australia; ^2^ Monash Department of Obstetrics and Gynaecology, Monash Health, Clayton, VIC, Australia; ^3^ Eastern Health Clinical School, Monash University, Box Hill, VIC, Australia

**Keywords:** hyperandrogenism, hyperinsulinemia, polycystic ovary syndrome, obesity, gestational diabetes (GDM), type 1 diabetes (T1D), type 2 diabetes, pregnancy

## Abstract

During pregnancy, the fetoplacental unit is key in the pronounced physiological endocrine changes which support pregnancy, fetal development and survival, birth and lactation. In healthy women, pregnancy is characterized by changes in insulin sensitivity and increased maternal androgen levels. These are accompanied by a suite of mechanisms that support fetal growth, maintain glucose homeostasis and protect both mother and fetus from adverse effects of pregnancy induced insulin and androgen excess. In pregnancies affected by endocrine, metabolic disorders such as polycystic ovary syndrome (PCOS), diabetes and obesity, there is an imbalance of beneficial and adverse impacts of pregnancy induced endocrine changes. These inter-related conditions are characterized by an interplay of hyperinsulinemia and hyperandrogenism which influence fetoplacental function and are associated with adverse pregnancy outcomes including hypertensive disorders of pregnancy, macrosomia, preterm delivery and caesarean section. However, the exact underlying mechanisms and relationships of the endocrine and metabolic milieu in these disorders and the impact they have on the prenatal endocrine environment and developing fetus remain poorly understood. Here we aim to review the complex endocrine and metabolic interactions in healthy women during normal pregnancies and those in pregnancies complicated by hyperinsulinemic disorders (PCOS, diabetes and obesity). We also explore the relationships between these endocrine and metabolic differences and the fetoplacental unit, pregnancy outcomes and the developing fetus.

## 1 Introduction

During pregnancy, pronounced physiological endocrine changes support maintenance of pregnancy, fetal development and survival, birth and lactation. Hormonal factors including progesterone, estrogens, androgens, and glucocorticoids are important during critical developmental windows of pregnancy (1). Increasingly, links between maternal endocrine metabolic conditions and pregnancy outcomes are recognized, with insulin resistance and hyperandrogenism central in these interactions. During normal progression of pregnancy, insulin resistance (IR) increases in the mother to provide energy for the growing fetus and (2) androgens increase, regulating key processes during pregnancy and parturition (3). These changes are balanced by pregnancy-specific mechanisms that are activated to maintain glucose homeostasis and to protect both the mother and fetus from pregnancy-induced insulin and androgen excess (4, 5).

Endocrine metabolic disorders such as polycystic ovary syndrome (PCOS), type 1 and type 2 diabetes (T1D, T2D), gestational diabetes (GDM) and maternal obesity are common and the prevalence of these conditions is rising globally, in line with increasing obesity. These conditions influence the endocrine environment and impact of the balance of beneficial and protective mechanisms in pregnancy. PCOS, the most common endocrinopathy in reproductive age women, affects 8 to 21% of this population. (6) PCOS is diagnosed (Rotterdam criteria) based on the presence of two of three features: oligo- or anovulation, hyperandrogenism (clinical or biochemical), and polycystic ovary morphology on ultrasound (7). Pre-existing diabetes is estimated to affect around 2% of pregnancies, doubling from 1990 to 2020 (8), while diabetes in pregnancy (DIP), either from pre-existing diabetes or GDM, affects approximately 16% of pregnancies globally (9). Overweight and obesity, characterized by an elevated body mass index (BMI) of 25 kg/m (2) and 30 kg/m (2) respectively, currently affects 26% and 25% of US women of reproductive age (20-39 years) (10). Globally, 18% and 20% of pregnant women start pregnancy overweight or obese and 47% gain gestational weight above that recommended by guidelines (11). These conditions are interrelated, with GDM prevalence closely associated with maternal obesity (12) and with PCOS (13).

According to the Barker hypothesis, the health and development of children is directly affected by fetal programming *in utero* during critical stages of development in early embryonic and fetal life (14). Critically, the presence of PCOS, diabetes or obesity is associated with higher rates of hypertensive disorders of pregnancy, macrosomia, pre-term delivery and caesarean sections, among other adverse impacts (15–19). These conditions may also lead to unfavorable long-term developmental programming in offspring and manifest as higher rates of metabolic disorders in adulthood, adverse cardiometabolic profiles and even mortality (16, 20). However, the exact underlying mechanisms and relationships between the endocrine and metabolic milieu in these disorders and the mechanisms and impacts they may have on the developing fetus are not fully understood.

A complex interplay of hyperinsulinemia and hyperandrogenism alters the endocrine milieu in pregnant women with PCOS, diabetes and obesity. In non-pregnant women with PCOS, insulin resistance (IR) and compensatory hyperinsulinemia contribute to hyperandrogenic traits as insulin facilitates androgen secretion (21). In turn, androgen excess itself facilitates metabolic dysfunction (21). This persists in pregnancy when women with PCOS are reported to have higher IR, insulin and androgens compared to women without PCOS (22–26). Systemic hyperinsulinemia in pregnancy also occurs with endogenous factors such as obesity (27), GDM (28) and T2D or exogenous factors (such as insulin use in type 1 diabetes or GDM) (29). Associations between maternal hyperinsulinemia and adverse neonatal outcomes are poorly understood, but some studies point to a role of hyperinsulinemia in macrosomia, neurological disorders and endothelial dysfunction in the neonate and impaired glucose tolerance in childhood (30, 31). These endocrine metabolic conditions are also characterized by hyperandrogenism. Fetal exposure to high androgen concentrations is associated with virilization (32–34), intra-uterine growth restriction (IUGR) (34, 35), placental differentiation (36), reproductive and metabolic dysfunction (37–42), adverse cardiac programming (43–45), and behavioral outcomes later in life (46–48). Hyperandrogenism is also associated with high levels of Anti-Müllerian hormone (AMH) in women with PCOS (49). AMH is a member of the transforming growth factor beta (TGFβ) family, produced by granulosa cells of the ovarian (pre-) antral follicles. Most non-pregnant women with PCOS exhibit higher levels of AMH than women without PCOS, with a positive correlation with androstenedione (A4) and testosterone (T) (49). Further, a positive correlation between AMH levels and HOMA-IR has been reported in non-obese women with PCOS (50). Elevated AMH concentrations have also been seen in prepubescent girls with T1D, suggesting a stimulatory effect of insulin therapy on granulosa cells (51). Recently, a number of cohort studies have reported elevated levels of AMH in pregnant women with PCOS versus controls (22, 23, 25, 52, 53), and positive correlations between AMH and maternal total T levels (53). Altered levels of AMH are associated with increased risk of miscarriage (53), lower live birth rates in women with PCOS undergoing assisted reproductive technology (54), milder forms of Mullerian anomalies (22), preterm delivery (55–57) and PCOS features in adulthood (53).

With the rising incidence of these endocrine metabolic disorders in pregnancy, an improved understanding of their impact on pregnancy and on the developing fetus and child is pertinent. Whilst this is a broad topic with other factors involved such as lipid metabolism, growth factors and cytokines, here we will focus on core hormonal factors linking endocrine and metabolic dysfunction - hyperinsulinemia, hyperandrogenism and AMH. This review aims to explore the complex endocrine and metabolic interactions in healthy women during normal pregnancies and those in pregnancies complicated by hyperinsulinemic disorders (PCOS, diabetes and obesity). We also explore the relationships between these endocrine and metabolic differences and the fetoplacental unit, the developing fetus and child health.

## 2 Endocrine and metabolic interactions in healthy pregnant women

During pregnancy, pronounced physiological endocrine changes occur to support fetal development and survival, birth and lactation. Effective exchange of nutritive and metabolic products is essential for intrauterine life. Although the endocrine systems are compartmentalized, processes within the fetal, placental and maternal compartments, complement each other, functioning as a unit that utilizes building materials from the maternal compartment for steroidogenic activities (58, 59). The fetus influences maternal adaptations, and thereby its own growth and development. Moreover, mechanisms involved in the regulation and metabolism of sex steroids during pregnancy could be dependent on adaptations in the maternal/placental compartment, determined by the fetal gender (60). Relative contributions of sex steroids shift from maternal ovaries and adrenals to the fetoplacental unit. The fetoplacental unit also acts as a barrier to some substances crossing to the fetus, with maternal hormones larger than 0.7 kDA barely passing the placenta. This ensures that the fetal endocrine environment is largely independent of maternal hormones. Whilst steroids are highly lipophilic and cross the placenta in both directions, most of them are metabolized en route (59). Although the placenta functions as a hypothalamic-pituitary-end organ-like entity, placental function is more complex.

In the sections below, we will describe the endocrine actions of the fetoplacental unit in relation to reproductive and steroid hormones and the impact of insulin, androgens and AMH on these hormones in healthy pregnant women.

### 2.1 Actions of the fetoplacental unit

Taking over from LH eight days after ovulation, hCG supports survival of the corpus luteum. A maximum level is reached at 8-10 weeks of gestation, then decreases to 10,000-20,000 IU/L by 18-20 weeks and remains at that level to term. Similar to the pituitary secretion of gonadotropins, inhibin and progesterone are inhibiting factors and estrogen and activin are enhancing factors of GnRH-hCG regulation. Maternal serum FSH concentrations are almost undetectable, and LH concentrations slowly decline until birth (3). Elevated levels of hCG in the second trimester are associated with miscarriage, small-for-gestational age infants, pre-eclampsia and preterm delivery (58).

#### 2.1.1 Progesterone

Progesterone prepares and maintains the endometrium to allow implantation, is important in maternal immunologic responses to fetal antigens and has a role in parturition. Initially, the corpus luteum is responsible for its production until about 10 weeks of gestation. After a transition period, the placental unit functions as the major source of progesterone synthesis and maternal circulating levels increase across pregnancy to reach a peak concentration (in order of 130 ng/ml) in the third trimester (59) whilst the fetal contribution is negligible (58). The precursor for progesterone derives from maternal cholesterol and production is therefore independent of uteroplacental perfusion or fetal wellbeing. Firstly, cholesterol is converted to pregnenolone by cytochrome P450scc (CYP11A1). Secondly, Pregnenolone is converted to progesterone by type 1 3β-hydroxysteroid dehydrogenase (HSD3B1) (59). The fetus uses progesterone to synthesize biologically important corticosteroids such as cortisol and aldosterone (58).

#### 2.1.2 Estrogens

Three forms of estrogen are produced in women: estrone (E1), estradiol (E2) and estriol (E3) (61). During pregnancy, E1 and E2 production is increased about a 100-fold, and E3 secretion by about a 1000-fold. This increase is due mainly to estrogen production in the placenta (59). Estrogens contribute to progesterone production, maternal cardiovascular adaptations, blood volume and uteroplacental blood flow. They also regulate genes involved in cholesterol supply to the placenta which is important for fetal and placental steroid hormone production (62). Additionally, estrogens exert effects on the developing fetus by maintaining intrauterine homeostasis, promoting maturation of fetal organs, regulating the fetal neuroendocrine system and regulating timing of parturition (63).

Human placental estrogen synthesis depends on DHEA and its sulfated form (DHEAS), produced from maternal androgens in the early months of gestation and derived from fetal androgens by the 20^th^ week of pregnancy (64). Further conversion of DHEAS to E1 and E2 requires four key enzymes. Placental sulfatases convert DHEAS into DHEA. Placental type I 3*β*-hydroxysteroid dehydrogenase (HSD)/*Δ*5*Δ*4isomerase (HSD3B1), converts DHEA into *Δ*4-androstenedione. Aromatase irreversibly converts *Δ*4-androstenedione into estrone (E1). Finally, E1 is converted into E2 by 17-*β*-HSD type 1 (HSD17B1) and then delivered to the maternal circulation (64). Placental E3 formation is carried out by 16a hydroxylation in the fetal liver (58), and then metabolized by placental sulfate, 3β-hydroxysteroid dehydrogenase, 17-hydroxysteroid oxidoreductase and maternal P450 aromatase to yield estriol (59). Moreover, the human fetal liver responds by increasing production of SHBG in order to bind estrogen in the circulation and limit levels of bioactive estrogens (65). These processes all ensure that biological effects of potent steroids are blocked. After birthing this ability disappears and estrogen concentrations decline rapidly in the postpartum period (3). Because estrogen production is dependent on fetal and placental steroidogenic cooperation, the amount of estrogen (especially estriol) present in the maternal blood or urine reflects fetal and placental enzymatic capability and, hence, well-being (58). In fact, Wan et al. reported lower levels of estrogen in preeclampsia and speculate that this reduction may be due to the impairment of placental function in preeclampsia (66).

#### 2.1.3 Activin and inhibin

Inhibin, produced by the placenta, shows increased levels during pregnancy (3). It peaks at 8 weeks gestation, then decreases (67), and increases again in the third trimester to 100-fold more than that during the normal menstrual cycle (3). Inhibin and estrogen account for the suppression of maternal gonadotropins during pregnancy (58). Activin A is also increased during pregnancy, with stable levels from 8 to 24 weeks and then increasing to a 100-fold of that during the normal menstrual cycle (58). Inhibin and Activin act as regulators within the placenta for the production of GnRH, hCG and steroids; inhibin is inhibitory and activin is stimulatory. Abnormal concentrations of Inhibin A have been associated with miscarriage, fetal growth restriction, gestational diabetes, and pre-eclampsia (68, 69). Elevated levels of Activin-A are associated with preeclampsia (70–72).

**Figure 1:**
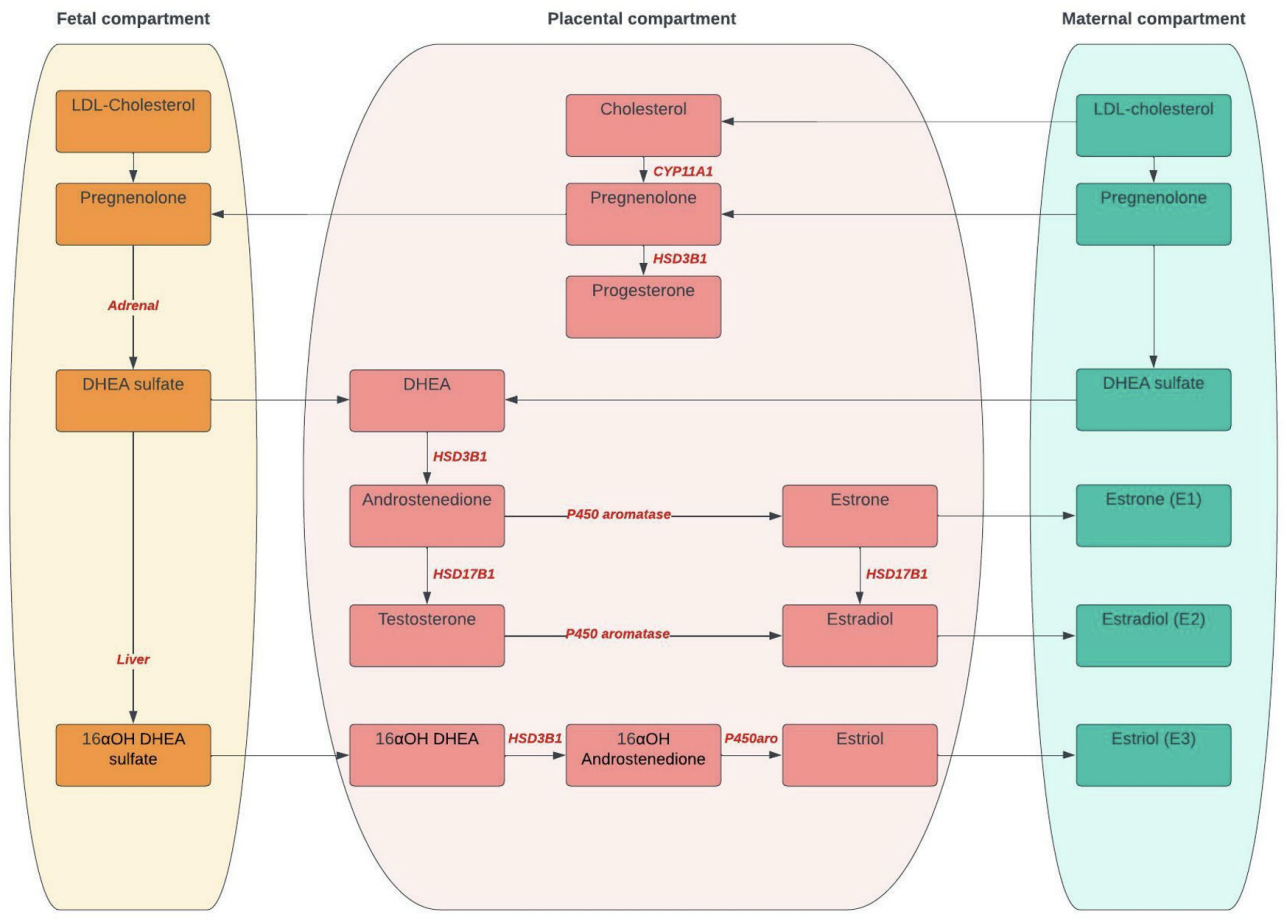
Steroid synthesis during pregnancy. CYP11A1, Cytochrome P450 side-change cleavage enzyme; HSD3B1, 3*β-*hydroxysteroid dehydrogenase 1; HSD17B1, 17-*β-*HSD type 1; DHEA, Dehydroepiandrosterone; 16αOH; 16α-hydroxydehydro.

A summary of steroid synthesis during pregnancy is provided in **Figure 1** with details in **Table 1**.

**Table 1:** Physiology of the feto-placental unit and the influence of metabolic factors in healthy pregnant women vs pregnant women with hyperinsulinemic disorders.

	Actions in healthy pregnant women	Actions in pregnant women with hyperinsulinemic disorders
GnRH	- Regulates placental steroidogenesis and release of prostaglandins and human chorionic gonadotrophin (hcG). GnRH secretion from the fetoplacental unit is controlled similarly to the hypothalamus and is increased by estrogen, activin-A, insulin and prostaglandins, and inhibited by progesterone and inhibin (58). - The GnRH receptor is highly expressed in the fetal adrenal gland as well, which shows another possible pathway by which the placenta can influence fetal adrenal function (58).	- Studies lacking
LH	- Slowly declines until birth (3)	- Studies lacking
FSH	- Almost undetectable (3)	- Studies lacking
Estrogens (E1, E2, E3)	- Contribute to progesterone production, maternal cardiovascular adaptations, blood volume, uteroplacental blood flow, cholesterol supply to the placenta, maintaining intrauterine homeostasis, maturation of fetal organs, timing of parturition, regulation of fetal neuroendocrine system - Estrone and estradiol production increased about a 100-fold, estriol secretion by about a 1000-fold. - Placental estrogen synthesis depends on DHEA (S) from maternal and fetal androgens	- E2 levels similar in PCOS vs non-PCOS (22, 73, 74) - E1 and E2 lower in T2D and GDM compared to healthy controls (75)
Progesterone	- Prepares and maintains endometrium, important in maternal immunologic responses to fetal antigens, role in parturition (58). - Produced as a result of placental-maternal cooperation (58) - Maternal circulating levels increase to high concentration in third trimester (59). - Used by the fetus to synthesize corticosteroids such as cortisol and aldosterone (58)	- Studies lacking
Inhibin B and Activin	- Produced by the placenta, increased levels during pregnancy (3). - Regulators for the production of GnRH, hCG and steroids; inhibin is inhibitory and activin stimulatory (58).	- Studies lacking
AMH	- Levels decrease as a result of hormonal suppression and inhibition of folliculogenesis (76, 77). - Relationship with other reproductive hormones in pregnancy have not been established	- Elevated in women with PCOS vs controls (22, 23, 25, 52, 53). - No significant differences between pregnant women with T2D/GDM vs without T2D/GDM (78, 79). - Elevated levels correlated with maternal total testosterone levels (22, 25, 49) - Elevated levels correlated with diminished placental metabolism of T to estradiol (53) - Elevated levels found in umbilical artery and vein in women with PCOS vs controls (22) - Impact of elevated levels include risk of miscarriage and preterm delivery (53, 55–57)
SHBG	- Increased production in order to bind estrogen and T (65).	- Lower levels among women with GDM in women with and without PCOS (27, 80–82). - Inverse association with fasting insulin and insulin resistance (82).
Insulin	- Insulin sensitivity declines throughout pregnancy as a result of placental hormones resulting in slightly elevated blood glucose levels to support the growing fetus (73–75). - Inhibits hepatic SHBG production (3).	- GDM is characterized by lack of insulin action, b-cell impairment and insulin resistance (83). - Higher insulin levels and HOMA-IR scores in women with PCOS and GDM (26) - Hyperinsulinemia precedes development of later gestation hyperglycemia and GDM (81, 82, 84–86) - Maternal and fetal hyperinsulinemia also associated with maternal obesity (87). - May directly stimulate androgen production or inhibit placental aromatase activity (24, 88). - Limited studies to confirm diagnostic thresholds for hyperinsulinemia in pregnancy (89) - Fetal hyperinsulinemia associated with macrosomia, neurological disorders, endothelial dysfunction and metabolic complications later in life (30, 31)
Androgens (DHEA, DHEA-S, A4, T and DHT)	- Rise of testosterone levels during pregnancy (3) as a result of decrease in metabolic clearance and adipose tissue aromatization (90, 91). - Effects on genital development and maintenance of pregnancy and initiation of parturition (92). - A4 levels peak in third trimester (3). Associated with fetal size, duration of gestation and onset of labor (93). - DHEAS major source of estriol production in fetal-placental unit (92). - Women and their fetus largely protected from androgen excess by SHBG and placental aromatization (4, 65).	- Elevated levels in pregnant women with PCOS (22–25, 94) and obesity (60). - Conflicting results in pregnant women with T2D/GDM (75, 95) - Decreased E2/T ratios in women with T2D and GDM (75). - Alterations of steroidogenesis in placenta or fetus may account for altered androgen levels (96). - Effects of fetal exposure to high androgen concentrations include virilization, IUGR, reproductive function including PCOS phenotypes, metabolic dysfunction later in life (97).

### 2.2 Metabolic factors influencing actions of the feto-placental unit

To understand the potential impacts of high levels of insulin, androgens and AMH, an understanding of their actions in normal pregnancies is critical. The sections below describe the normal actions of insulin, androgens and AMH and their relation to the hormones described in the previous sections.

#### 2.2.1 Insulin

Depending on the requirements of pregnancy, insulin sensitivity shifts. At 12–14 weeks’ gestation, insulin sensitivity is somewhat increased to promote the uptake of glucose into adipose stores in preparation for the energy demands later in pregnancy. However, insulin sensitivity then declines throughout pregnancy as a result of local and placental hormones, including estrogen, progesterone, leptin, cortisol, placental cytokines (especially TNF-α), placental lactogen (hPL) and placental growth hormone that promote IR (98, 99). This results in slightly elevated blood glucose levels as a means to provide energy to support the growing fetus. The state of IR also promotes endogenous glucose production and breakdown of fat stores with further increase in blood glucose concentrations (100). Hypertrophy and hyperplasia of pancreatic β-cells and increased glucose-stimulated insulin secretion compensate for these changes to maintain glucose homeostasis, as shown in animal studies (101). Insulin has been found to inhibit hepatic SHBG production (3) and, low SHBG concentrations might even reflect IR better than fasting glucose or insulin levels (102, 103).

#### 2.2.2 Androgens

The fetoplacental unit, the adrenals and potentially adipose tissue have been assumed to be responsible for maternal androgen levels in pregnancy (90, 97). During normal progression of pregnancy, levels of total testosterone (T) increase by 70%, with peak levels in the third trimester of pregnancy (3). Among factors contributing to the rise of T are the decrease in metabolic clearance of T (91) and adipose tissue aromatization (90). Among others, T exerts effects on genital development during early gestation (3) and on the maintenance of pregnancy and initiation of parturition (92). Maternal serum A4 concentrations start to increase in the later part of pregnancy, with peak levels in the third trimester (3). A4 levels have been associated with nausea, fetal size, duration of gestation and onset of labour (93). DHEAS plays a role in cervical ripening through activation of collagenase activity induced by the enhanced conversion to E2 (104). It is also a major source of E3 production in the fetal-placental unit (92). It has been shown to decline during pregnancy (105). However, recent studies suggest a more fluctuating pattern of DHEAS throughout gestation (3).

Pregnant women and their infants remain largely unaffected by the changes in androgen concentrations due to pregnancy-specific mechanisms that are activated to protect both the mother and fetus from this androgen excess. The increased levels of SHBG bind T, while the cytochrome P450 aromatase enzyme converts androgens into E2. The fetal liver subsequently converts E2 to E3, which is then excreted in maternal urine. Placental aromatization is so efficient, that little androgen presented to the placenta escapes (4). It has been proposed that the increase in SHBG during pregnancy protects the fetus from exposure to maternal androgens, however, reciprocally, maternal SHBG may protect mothers from androgens originating from the fetus (65). This has been demonstrated in a clinical case report that showed profound and transient maternal virilization resulting from very low plasma SHBG, whereas the twin daughters did not show any evidence of excessive androgen exposure (106). Something similar has been demonstrated in patients with a fetus with a defective P450 aromatase gene, which resulted in a spillover of fetal androgens into the maternal circulation (107). This highlights the importance of SHBG and placental aromatase in protecting the mother from fetal androgens.

#### 2.2.3 Anti-Müllerian hormone

During pregnancies of women without hyperinsulinemic disorders, AMH levels significantly drop during the course of pregnancy and immediately after birth, (first trimester: 1.69 ng/ml (IQR 0.71-3.10), second trimester: 0.8 ng/ml (IQR 0.48-1.41), third trimester: 0.5 ng/ml (IQR 0.18-1.00)) but subsequently increases over the first four days postpartum (76). This decrease throughout the three trimesters of pregnancy most likely results in the inhibition of folliculogenesis in pregnancy (78). Hormones such as estrogen may regulate AMH production (77), however some studies have not found associations between AMH levels and E1 in pregnancies in women with or without T2D and GDM (78, 108). The relationship between other reproductive hormones (e.g. LH, activin, inhibin) and AMH production has not been established. Although possible involvement of these factors in regulating gravid AMH levels cannot be excluded, other factors, such as Follistatin (109) may also regulate AMH levels during pregnancy.

In summary, in normal pregnancies, insulin sensitivity declines as a result of local and placental hormones resulting in slightly elevated blood glucose levels to support the growing fetus, with a concomitant inhibition of hepatic SHBG production. Androgen levels increase due to decreased metabolic clearance and adipose tissue aromatization to regulate key processes during pregnancy and parturition. Mechanisms such as altered SHBG that binds T and conversion of androgens into E2 by cytochrome P450 are in place to protect both the mother and fetus from androgen excess. Levels of AMH decrease as a result of hormonal suppression and inhibition of folliculogenesis. Relationships between AMH and other reproductive hormones in pregnancy are not extensively studied.

## 3 Hormonal and metabolic interactions in pregnant women with hyperinsulinemic disorders

The interrelationships between hyperinsulinemia, androgen excess, and AMH are potentially reflected in pregnancies complicated by hyperinsulinemic disorders. The preconception state of hyperinsulinemia and/or androgen and AMH excess might drive the mechanisms underlying fetoplacental endocrine dysfunction. However, because hyperinsulinemic conditions including PCOS, diabetes (GDM, T1D and T2D), and obesity often appear concurrently, no one condition explains the altered endocrine milieu and eventual adverse maternal and neonatal outcomes in these women. The following section aims to assess the endocrine and metabolic factors, focusing on insulin, androgens and AMH (and their interactions) in women with PCOS, diabetes and obesity and how they impact the fetoplacental unit.

### 3.1 Actions of the fetoplacental unit and the influence of metabolic factors in women with hyperinsulinemic pregnancies

Clinical studies on placental GnRH, hCG, estrogens, progesterone, activin and inhibin in women with hyperinsulinemic conditions in pregnancy are scarce. Studies have reported differences in some hormones in pregnant women with and without PCOS, but do not specifically compare the fetoplacental unit between these groups or address interactions between androgens, estrogens, AMH and pituitary hormones in the mothers and the fetus (22). Maternal E2 levels appear similar in women with and without PCOS (22, 73, 74). A recent study found that E1 and E2 levels were lower in women with T2D and GDM than in healthy controls during the second half of pregnancy (75). Data on cord blood estrogens show conflicting outcomes. Lower E1 concentrations were found in cord blood from girls and women with PCOS compared with controls, but there were no differences in E2 and E3 in these studies (74, 88). Anderson et al. (73) reported decreased E2 levels in female PCOS cord blood samples compared with controls, and lower E2 levels have been noted in cord blood compared to maternal levels (22, 110). Few studies report on estrogens in relation with other reproductive and metabolic hormones.

#### 3.1.1 Hyperinsulinemia

Whereas increased IR during pregnancy is an expected physiological state, complications arise when these changes are augmented by pre-existing hyperinsulinemia and related metabolic dysfunction. When there is a relative lack of insulin action in body tissues to overcome IR, GDM ensues (83). Critical to the pathophysiology of GDM is β-cell impairment aggravated by tissue IR during pregnancy. These impairments usually exist prior to pregnancy and can be progressive. Women with a history of GDM were almost 10 times more likely to develop T2DM than those with a normoglycemic pregnancy with pooled cumulative incidence estimated to be around 16% for studies with more than 10 years of follow up (111). Reduced insulin-stimulated glucose uptake further contributes to hyperglycemia which overburdens the β-cell, producing an additional insulin response and worsening β-cell dysfunction (5). IR, which includes a failure of insulin signaling, further exacerbates β-cell dysfunction (5).

The mechanisms underlying GDM are thought to be similar to other disorders of insulin sensitivity such as T2DM, prediabetes, obesity and PCOS. De Wilde et al. (26) demonstrated that in 22 women with PCOS and GDM, insulin levels and homeostasis model assessment of insulin resistance (HOMA-IR) scores were higher before conception and at each sampling point in pregnancy compared to women with PCOS who did not develop GDM, independently of BMI. Insulin sensitivity in women with PCOS who develop GDM is potentially not as high as in normal glucose-tolerant women in the first trimester resulting in hyperinsulinemia to keep glucose concentrations within normal ranges. Additionally, insulin levels and HOMA-IR levels were already significantly increased prior to conception in the GDM group compared with the non-GDM group. These findings support the hypothesis that the risk of developing GDM is already present early in pregnancy. A growing body of evidence suggests that hyperinsulinemia in early gestation precedes later gestation hyperglycemia and GDM development (89, 112–114). Associations have also been found between maternal obesity and maternal and fetal hyperinsulinemia (87).

Insulin interacts with other important metabolic markers. High levels of insulin may contribute to fetal hyperandrogenism exposure by directly stimulating androgen production (24). Furthermore, insulin, Insulin-like growth factor 1 (IGF-I) and IGF-II have been shown to inhibit placental aromatase activity (88). This is important, given the exacerbated IR and increased levels of insulin in pregnant women with PCOS. In turn, a decrease in P450 aromatase activity has been described in GDM and pre-eclampsia (115, 116). Importantly, a recent meta-analysis of prospective studies found an inverse association of SHBG with fasting insulin and IR (80). SHBG levels were lower among women who subsequently developed GDM compared to those who did not, independent of adiposity (80). Similarly, lower preconception SHBG concentrations are also associated with GDM in women with PCOS (81, 82). The study by de Wilde et al. (26) also demonstrated that in 22 women with PCOS and GDM, SHBG levels were significantly lower before conception and in the second trimester. Low plasma SHBG in pregnancy was also associated with hyperandrogenism (106). We therefore hypothesize that like outside pregnancy, hyperinsulinemia in these disorders and subsequent direct stimulation of androgen production, and inhibition of SHBG (that binds androgens), contributes to hyperandrogenism. In pregnancy, inhibition of placental aromatase activity is likely to exacerbate this phenomenon.

Hyperinsulinemia in pregnancy may impact maternal and fetal health. Routine assessments and standardized diagnostics of hyperinsulinemia in pregnancy are lacking. Therefore, associations between pathological hyperinsulinemia and maternal and neonatal outcomes are not well understood. However, it is proposed that hyperinsulinemia has effects on maternal hemodynamic adaptations, potentially increasing the risk of pre-eclampsia, stillbirth and intrauterine growth restriction (84). Studies have reported increased insulin in cord blood of babies born to mothers with T2D and GDM compared to controls (85, 86). Fetal hyperinsulinism is associated with increased risk of later metabolic complications (30). Further, some studies point to a role of hyperinsulinemia in fetal development such as macrosomia, neurological disorders, endothelial dysfunction in the neonate (31), offspring hyperinsulinemia and cardiac hypertrophy (84). North et al. (89) hypothesize that hyperinsulinemia in women with “borderline” glucose intolerance may explain pivotal observations from The Hyperglycemia and Adverse Pregnancy Outcome (HAPO) cohort that showed an increased risk of fetal overgrowth, primary caesarean delivery, elevated cord-blood Connecting peptide (C-peptide) and neonatal hypoglycemia in mothers with mild-to-moderately elevated glucose levels (19). Importantly, metformin use in pregnancy might enhance insulin sensitivity, reduce insulin resistance and fetal hyperinsulinemia and in turn, reduce neonatal adiposity (117). However, it’s use in pregnancy continues to be investigated to determine efficacy and safety for preventing or treating GDM (118).

Human data on the effects of exogenous insulin use in pregnancy in relation to other reproductive factors or pregnancy outcomes is scarce. Insulin does not cross the placenta and in animal studies, exogenous insulin has no effects on embryo or fetal development (119). However, insulin receptors are expressed on the placenta and may be a means through which insulin mediates effects on the fetus through action on the placenta (84). A recent meta-analysis compared outcomes between women with GDM with insulin use, GDM without insulin use and no GDM (120). In studies with no insulin use, when adjusted for confounders such as BMI, women with GDM had increased odds of caesarean section, preterm delivery, low one-minute Apgar scores, macrosomia and infant born large for gestational age compared with women without GDM. In studies with insulin use, when adjusted for confounders, women with GDM had increased odds of having an infant born large for gestational age, with respiratory distress syndrome, neonatal jaundice, and/or requiring admission to the neonatal intensive care unit compared to women without GDM (120). Furthermore, an increase in placental weight in women with GDM that used insulin versus women with GDM that were controlled with diet and exercise only was observed (121). In pregnant women with T1D, the use of insulin lispro was associated with an increased risk of macrosomia (122). Importantly, a high incidence of macrosomia was found despite overall good glycemic control, as assessed by HbA1c. It was proposed that the acute pulsatile rise and fall of maternal blood glucose levels which occur after food intake (hyperglycemia) and insulin treatment (hypoglycemia), respectively, might result in increased fetal release of endogenous insulin leading to increased placental weight and macrosomia (121, 122). However, clinical implications of hyperinsulinemia and links to perinatal and neonatal outcomes remain poorly understood. There is limited research to confirm diagnostic thresholds for hyperinsulinemia in to insulin metabolism become potentially harmful are needed (89).

Overall, studies show relative hyperinsulinemia during pregnancy in women with PCOS GDM, and maternal obesity, compared to healthy pregnancies without these conditions. Hyperinsulinemia contributes to fetal hyperandrogenism exposure by stimulating androgen production, inhibiting placental aromatase activity and inhibiting SHBG. Hyperinsulinemia may also increase risks of PE, stillbirth, IUGR, macrosomia, and neurological disorders, endothelial dysfunction and later metabolic complications in the offspring. Finally, similar to the exogenous action of insulin on androgen excess in women with T1D, we hypothesize that the use of insulin in women with GDM (with or without PCOS) might also aggravate hyperinsulinemia in pregnancy; however, this phenomenon and its potential complications await further study.

#### 3.1.2 Hyperandrogenism

The adaptations which occur in pregnancy to protect both the mother and fetus from pregnancy-induced androgen excess might not be sufficient in pregnant women with underlying hyperinsulinemic pregnancies, as evidenced by elevated levels of androgens in pregnant women with PCOS. Several studies found elevated serum androgen levels, including T (22–25, 94), A4 (23, 24, 94), DHEAS and free androgen index (FAI) (24, 25, 94) in pregnant women with PCOS. This may be a potential source of fetal androgen excess and induce effects on the development of the fetus, even if virilization of a female fetus is not observed (24). Results regarding T and GDM are conflicting. Some report that women who develop GDM have significantly higher T concentrations compared with controls (95), while others demonstrated hyperandrogenemia in pregnant women with T2D, but not in GDM (75). Additionally, decreased estrogen and E/T ratios were found in women with T2D and women with GDM (75). In the latter study, BMI and T levels were positively associated in the T2D and GDM groups (75). Maternal obesity has also been found to be associated with elevated maternal serum T concentrations (60).

In relation to the impact of androgen excess on fetal health, some studies have used cord blood to investigate the intrauterine fetal environment in women with PCOS (22, 73, 74, 88, 123, 124). Levels of DHEA and DHEAS in fetal cord blood were generally not associated with PCOS (73, 74, 88, 95), but studies regarding T and A4 have produced conflicting results. A recent meta-analysis of seven studies (n= 570) found no significant differences in cord blood T levels between women with and without PCOS, irrespective of neonatal sex (125). The authors suggest that T may be quickly degraded and converted by placental aromatase into E2 when passing through the placenta. However, cord blood A4 levels in female newborns were significantly lower in PCOS than in the control group (125). As explained in section 2, cord blood androgens are derived from both fetal adrenal as well as placental steroidogenesis. The fetal adrenals produce DHEAS *in utero*, which is transformed into A4, T, and E2 by the placenta (126). Alterations of steroidogenesis in the fetus or an abnormality in placental steroidogenesis could potentially account for the decreased A4 levels (125). Similarly, Kelly et al. (96) proposed that maternal androgens may exert a programming effect on placental and/or fetal steroidogenesis to alter androgen levels within the fetal-placental unit. This concept is supported by Maliqueo et al. (88), wherein placental tissue from women with PCOS had increased 3*β*-hydroxysteroid dehydrogenase 1 enzymatic activity and decreased aromatase activity compared with non-PCOS controls inducing accumulation of androgenic substrate. This is in line with findings of lower E1 and E2 serum levels in women with GDM and lower E2/T ratios suggesting a lower conversion of T to estrogens (75).

Despite the fact that maternal androgen excess was not associated with elevated T concentrations in cord blood, hyperandrogenism in pregnancy is clinically relevant given the numerous reports, both from animal models and clinical studies, on the adverse effects of fetal exposure to high androgen concentrations. Importantly, umbilical cord blood is only a representation of the end of pregnancy rather than the entire gestation period. The effects of fetal exposure to high androgen concentrations have been reviewed in detail elsewhere and include virilization (32–34), IUGR (34, 35), placental differentiation culminating in low birth weight (36), reproductive function including PCOS like phenotypes (37–39), metabolic dysfunction (37, 39–42), adverse cardiac programming (43–45), and behavioral outcomes later in life (46–48).

To what extent high levels of androgens directly impact maternal and fetal health is not clear, given that androgens are converted to estrogens. In animal studies, examining direct effects of DHT, a nonaromatizable androgen, was associated with decreased placental weight and cotreatment of T with an androgen antagonist prevented placental changes, suggesting placental changes were mediated at least in part by androgenic action (96). Additionally, evidence from studies in rhesus monkeys indicates that gestational T and DHT treatment induces maternal hyperinsulinemia and insulin resistance in addition to elevating circulating androgen levels in both maternal and fetal compartments (42, 127). This may result in reprogramming of insulin target tissues in offspring such as liver and adipose tissue, leading to hyperglycemia in adulthood (128). As such, effects of hyperandrogenism on fetal development and pregnancy outcomes could be mediated *via* insulin and further altered by the impact of hyperinsulinemic states. Importantly, the inhibitory action of insulin and AMH on placental aromatase action might contribute to changes in sexual steroids and insulin levels during pregnancy. Placental steroidogenesis possibly follows different pathways in hyperinsulinemic pregnancies, which results in a different ratio of E/T fractions during gestation and in offspring.

In summary, studies have found relatively higher levels of androgens in pregnant women with PCOS and obesity, whereas this is less clear in T2D and GDM. Reports of cord blood levels of androgens are scarce and conflicting. Different androgen profiles within the fetal-placental unit may be reflective of abnormalities of steroidogenesis in the fetus or placenta. Hyperandrogenism in pregnancy is associated with adverse short- and long-term neonatal outcomes. Rather than a function of direct androgenic action only, effects from hyperandrogenism on fetal development and pregnancy outcomes are potentially mediated by insulin and further altered by hyperinsulinemic states.

#### 3.1.3 Anti-Müllerian hormone

As described in section 2, AMH levels decrease successively during pregnancy, mostly between the first and second trimester. Several studies show elevated levels of AMH during pregnancy in women with PCOS versus controls (22, 23, 25, 52, 53). In fact, high AMH levels in women with PCOS are maintained throughout the entire pregnancy (23). However, studies have not found significantly different AMH concentrations between women with diabetes (T2D; GDM) and healthy pregnant women (78, 79). In relation to obesity, studies found that AMH was negatively associated with maternal BMI (22, 108, 129).

Interactions between AMH and other hormones in pregnancy have recently been investigated by Tata et al. (53) who measured AMH in a cohort of 66 pregnant women with PCOS and 63 control women, at gestational week 16-19. AMH was significantly higher in pregnant lean women with PCOS compared with the control group but not in obese women with PCOS versus obese control women. Furthermore, AMH was significantly higher in lean women with PCOS who had hyperandrogenism than in those without. No differences between obese women with PCOS with and without hyperandrogenism were detected, potentially as the impact of obesity may negate PCOS effects on AMH. An animal model that treated pregnant mice with AMH resulted in maternal neuroendocrine-driven T excess and diminished placental metabolism of T to E2. Authors suggest that this could be a viable route by which maternal and placental T can be transferred to the human fetus (53). Although not extensively studied, lower levels of E1 and E2 in pregnant women with T2D and GDM and in female cord blood of women with PCOS support this suggestion, and could be reflective of increased AMH levels leading to decreased placental metabolism of T, however a direct relationship has not been established (73, 74, 78, 88).

Several other studies report a positive correlation between elevated AMH levels and maternal total T levels (both in women with PCOS as well as controls) (22, 23, 25). It is thought that the ovaries may contribute to maternal serum concentrations of T. In addition, high AMH may influence placental T production. This is in line with findings in prenatal AMH-exposed mice, in which AMH exposure was associated with elevated T levels in the dams and offspring. Treatment of pregnant mice with AMH resulted in a masculinization of the exposed female fetus and PCOS-like phenotypic traits in adulthood (reproductive and neuroendocrine) (53). Lastly, although studies in non-pregnant adolescents with PCOS show a positive association between serum AMH and HOMA-IR, no significant associations of AMH with insulin, HOMA-IR or IGF-1 have been observed in pregnant women (78, 108).

Elevated levels of AMH potentially impact the developing fetus. Studies have found elevated serum AMH levels in the umbilical vein at time of delivery in newborns of women with PCOS compared with healthy controls (110). Importantly, higher levels of AMH were observed in neonates born from women with PCOS and maternal hyperandrogenism or maternal BMI higher than 30 kg/m^2^ (110). On the other hand, no significant associations were observed between maternal AMH concentrations and AMH in female offspring (110), whereas AMH was higher in male fetuses, than in the mother (22). This suggests that AMH concentrations in umbilical cord blood might differ from that in maternal serum because there is no passage of AMH from fetus to mother (22, 110). Instead, elevated AMH concentrations could be a result of placental passage from the mother and increased AMH production by the fetal compartment. Detti et al. (22) hypothesize that genetic inheritance of PCOS by the fetus causes increased AMH production by the embryonal/fetal granulosa and Sertoli cells.

Studies investigating associations between levels of AMH and pregnancy outcomes are scarce. One study showed increased risk of miscarriage in association with prenatal exposure to AMH (53). Further, high serum AMH levels were associated with lower live birth rates in women with PCOS undergoing assisted reproductive technology (54). Studies have reported no association between maternal AMH and infant birthweight (25, 79, 129, 130). Increased incidence of PCOS is observed in women with sub-separate uteri. Detti et al. (22) hypothesize that higher AMH concentrations could contribute to development of milder forms of Mullerian anomalies by delay/arrest in Mullerian duct fusion and inner wall reabsorption (22, 110). AMH may also be associated with preterm delivery (55–57). Kaing et al. (57) examined women with PCOS and high AMH who conceived after ovulation induction, and found that 62% of women who delivered preterm had AMH levels above the 75^th^ percentile. Women with PCOS who delivered preterm had notably higher AMH than their term counterparts (11.1 vs 5.4 ng/mL). Similarly, a recent retrospective cohort study with patients with PCOS who had undergone IVF/ICSI showed that for patients with a BMI ≥ 24 kg/m (2) plus serum AMH > 6.45 ng/ml (75^th^ percentile), the risk of preterm birth was 2.1 times that in the AMH <2.71 ng/ml group (55).Associations with other maternal and perinatal outcomes remain uncertain. Most existing studies do not show statistically increased risk of other adverse maternal and perinatal outcomes in women with elevated AMH levels (25, 55, 130). Prenatal AMH treatment in mice resulted in PCOS features in adulthood: hyperandrogenism, LH elevation, sporadic ovulation, and fertility defects. However, these mice did not show weight alterations, leading to the assumption that prenatal AMH could predispose to the lean PCOS phenotype in adulthood (53).

In summary, elevated levels of AMH during pregnancy have been associated with T excess, and diminished metabolism of T to E2. However, associations between AMH and insulin or HOMA-IR in pregnancy remains to be determined. Elevated AMH may be associated with preterm birth in women with PCOS, however associations with other maternal and neonatal outcomes remain uncertain.

## 4 Limitations and future directions

While an abnormal prenatal endocrine milieu could possibly reflect a deranged feto-placental unit in women with hyperinsulinemic disorders, studies specifically addressing relationships and associations between insulin, androgens, pituitary hormones, estrogens, AMH and placental steroidogenesis are scarce. Studies are often cross-sectional in design, do not adjust for confounding factors such as nutrition and physical activity and longitudinal studies on maternal hormones do not always include early pregnancy samples. Furthermore, they are often limited by small sample sizes and to increase the power, the conclusions drawn from these studies need to be further studied with larger sample sizes. Data regarding subsequent alterations in the intrauterine environment, often studied in cord blood have produced conflicting results and overall evidence is scarce. Hormonal dysregulation in women with PCOS, diabetes or obesity may impact placental development and function. However, placental studies (looking at macroscopic and microscopic changes) in pregnancies impacted by these conditions are limited.

In order to study the interrelationships between insulin, androgens and AMH, investigating placental tissue could be an important additional mode of research.

Hyperinsulinemia in pregnancies affected by PCOS, obesity or diabetes seems largely a result of pre-existing hyperinsulinemia and related metabolic dysfunction paired with IR in pregnancy exacerbating B-cell dysfunction. However, further research is warranted on the factors that promote IR more than the physiological state of IR hPL for example, contributes to IR and secretion of insulin. Pregnancies affected by obesity and diabetes show altered hPL levels: lower concentrations have been shown in obesity and increased levels in GDM. Disruptions in hPL are also thought to be associated with an increased prevalence of placental dysfunction and increased fetal growth (131). Likewise, TNF-α, a proinflammatory cytokine produced by the placenta and other placental adipokines (e.g. Chemerin, Apelin, Omentin) also likely play a role in development of IR in pregnancy (131). In order to understand the pathways by which IR occurs, factors associated with IR in women with hyperinsulinemic pregnancies should be further explored.

Studies of diagnostic thresholds of insulin levels in pregnancy are lacking. Therefore, in order to study associations between pathological hyperinsulinemia and maternal and neonatal outcomes, criteria need to be established to determine when normal hyperinsulinemia in pregnancy results in levels that result in clinically relevant adverse outcomes. In addition, data on insulin levels and IR in pregnancies affected by metabolic and endocrine disorders is notably lacking. Longitudinal measurements of insulin and IR in pregnancy would be beneficial. The possible associations between maternal hyperinsulinemia and offspring health highlight the need for further study into the interactions and mechanisms between maternal insulin and fetal development and health through pregnancy, post birth and into childhood.

Whilst studies show that hyperinsulinemia is likely to result in decreased levels of SHBG (leading to increased free T), direct stimulation of androgens and inhibition of placental aromatase activity, future research is needed to substantiate associations between insulin levels in pregnancy and the maternal and prenatal endocrine milieu. Human association studies of the various hyperinsulinemic states, coupled with animal models that ablate variables of interest could help clarify these links. Furthermore, the use of insulin in women across a range of hyperinsulinemic disorders in pregnancy, the potential concurrent worsening of hyperinsulinemia in pregnancy, related hyperandrogenism and its potential complications have not been studied previously.

The effects of hyperandrogenism in pregnancy on developmental programming have been widely studied. However, questions regarding the pathways by which androgens impact maternal and fetal health remain unanswered. Whilst evidence is clear regarding the contribution of hyperinsulinemia on hyperandrogenism, few studies examine the effects of androgens on insulin and insulin sensitivity. Observational studies of various hyperandrogenic states coupled with further explorations using animal models will enhance understanding of how hyperandrogenism interacts with other variables such as estrogens, AMH, insulin and potential other markers in pregnancy.

The hyperandrogenic phenotype is likely to be underreported in maternal samples as observational studies do not always include a full assessment of the different androgens. This will influence interpretation of gestation AMH levels across PCOS phenotypes. Future research, with bigger sample sizes and full assessment of hyperandrogenism is warranted to confirm elevated levels of AMH during pregnancy between groups, to support the hypothesis that high AMH levels lead to decreased placental metabolism of T and to assess associations between AMH and insulin and HOMA-IR. Animal studies could explore what contributes to higher levels of AMH in pregnancy. In addition, the physiologically low AMH levels observed during the second half of pregnancy may require more sensitive tests to find a significant difference between groups under study. Previous studies assayed serum AMH using the widely clinically used assay AMH/MIS ELISA kit (Immunotech-Beckman, Marseilles, France) (75, 79). Due to a lower threshold of detection, a newer commercially available AMH enzyme-linked assay (pico AMH ELISA, AnshLabs) might be more appropriate for the measurement of low AMH concentrations (132). Further, studies are needed to investigate associations between high AMH levels and pregnancy and fetal and offspring health and to elucidate the potential actions of high AMH on the maternal and placental adaptations in pregnancy. Reports so far show no conclusive results and the polycystic ovarian morphology, reflected by high AMH levels, might not drive mechanisms behind adverse outcomes in pregnant women with PCOS.

Other factors important for pregnancy, birth, lactation, child development and survival, such as hCG, activin, inhibin, hPL and placental cytokines have not been widely studied in relation to hyperinsulinemic pregnancies but might be potential markers relevant to explain altered hormonal states in pregnancies affected by hyperinsulinemic disorders. For example, while Inhibin and Activin act as regulators within the placenta for GnRH, hCG and steroids, to our knowledge no reports are currently present on their actions in hyperinsulinemic pregnancies. Additionally, hyperinsulinemia could stimulate production of leptin which could amplify inflammation (133).

Interestingly, upon analyzing T and P450 aromatase in normal weight and obese women, Maliqueo et al. (2017) only found an increment of T and lower P450 aromatase in obese women with male fetuses. The authors therefore suggest that regulation and metabolism of sex steroids in pregnancy could be dependent on maternal/placental adaptations determined by fetal gender (60). In relation to pregnancies affected by hyperinsulinemic disorders, future research is warranted to understand the mechanisms by which fetal gender may impact placental function.

## 5 Conclusions

This comprehensive review compares physiological changes in normal pregnancies to those in women with pre-existing hyperinsulinemic conditions including PCOS, diabetes and obesity. We highlight how the maternal, placental and fetal compartments are separate, but functionally complement each other in healthy pregnancies. Yet in pregnant women with pre-existing hyperinsulinemic disorders, it appears that abnormal levels of insulin, androgens, estrogens and AMH are reflective of a deranged fetoplacental unit, ultimately leading to adverse pregnancy and neonatal outcomes. Hyperinsulinemia in these disorders and subsequent direct stimulation of androgen production, inhibition of SHBG and inhibition of placental aromatase activity, contributes to hyperandrogenism. Effects of hyperandrogenism could be mediated *via* insulin and altered placental steroidogenesis. Elevated levels of AMH contribute to T excess, and diminished metabolism of T to E2. With the high prevalence of hyperinsulinemic endocrine metabolic conditions including PCOS, diabetes and obesity in pregnancy, more research, including longitudinal sampling, and consideration of severity of hyperinsulinemia and interaction across these linked conditions is needed. A focus on placental and fetal steroidogenesis and hormonal interactions in these conditions is important. Finally, further study on the impacts of exogenous insulin administered in women with T1D, T2D and GDM, is needed, in relation to important endocrine and metabolic markers, and impact on fetal health.

## Author contributions

AN collated and reviewed the literature and wrote the first draft of the manuscript. AM, JB, and HT reviewed and edited the manuscript. All authors provided intellectual input in line with the ICMJE criteria for authorship and have approved the final version for publication.
